# Simultaneous bilateral versus unilateral total hip arthroplasty: Pain and physical function in a one- and five-year follow-up - retrospective patients record study

**DOI:** 10.1186/s12891-023-06743-w

**Published:** 2023-07-25

**Authors:** Leena Ristolainen, Jyrki Kettunen, Jouni Lohikoski, Hannu Kautiainen, Mikko Manninen

**Affiliations:** 1 Orton Orthopaedic Hospital, Tenholantie 10, Helsinki, 00280 Finland; 2 Arcada University of Applied Sciences, Jan-Magnus Janssonin aukio 1, Helsinki, 00550 Finland; 3 Primary Health Care Unit, Kuopio University Hospital, P.O. Box 100, Kuopio, FI 70029 KYS Finland

**Keywords:** Total hip arthroplasty, THA, Bilateral THA, Unilateral THA, Physical function

## Abstract

**Background:**

Total hip arthroplasty (THA) decreases pain and improves function in patients with osteoarthritis. In some cases, both hips have been operated simultaneously. Our aim was to report patients’ pain and physical function after one- and five-years post-operatively among patients who underwent unilateral THA and those who underwent bilateral THA at the same time in one orthopaedic hospital in Finland.

**Methods:**

The study group consisted of 488 patients retrospectively selected patients from a single centre; 421 of them underwent unilateral THA and 67 underwent simultaneous bilateral THA. The patients had two clinical examinations one and five years postoperatively. Systematic data about pain and physical function were collected using the scaled Orton Hip Score (sOHS). Register data on revisions and mortality events were from the Finnish Institute of Health and Welfare.

**Results:**

At the one-year follow-up, total sOHS was improved remarkably from the preoperative situation, both in the unilateral THA (age and gender adjusted mean improvement 42 points (95% CI: 40 to 44, p < 0.001) and in the bilateral THA groups (age and gender adjusted mean improvement 45 [95% CI: 41 to 49], p < 0.001), with no group differences after five-years of operation (age and gender adjusted p = 0.19). Total sOHS was statistically higher in the bilateral THA compared to the unilateral THA after one year (98 vs. 95, p < 0.001) and five years (97 vs. 95, p = 0.003) of operation.

**Conclusions:**

Patients in unilateral THA and bilateral THA groups had increased their physical function, and pain had decreased after one-year follow-up of the primary THA operation, and condition remained after five years of operation. At follow-ups, patients who underwent bilateral THA had slightly better physical function compared to patients who underwent unilateral THA at follow-up; however, this difference had no clinical relevance.

**Supplementary Information:**

The online version contains supplementary material available at 10.1186/s12891-023-06743-w.

## Background

Total hip arthroplasty (THA) is a common orthopaedic procedure. In Finland, approximately 9600 surgeries were performed in 2020. A higher proportion of THA patients are women (57%) and over 55 years of age [1]. Several reports have indicated that THA improves physical function and the patient’s health-related quality of life [2–4]. In 1976, Ritter and Randolph described the differences in functional outcome, operation time, and blood loss between patients who underwent simultaneous bilateral THA (later bilateral THA) and those who underwent unilateral THA [5]. Since then, the advantages and disadvantages of comparing these procedures have been discussed. Many studies have compared the length of stay at the hospital,mortalities, and complications between bilateral THA and unilateral THA; cost comparison studies have also been conducted [6–10]. Patients who underwent bilateral THA were on average younger than those who underwent unilateral THA. However, many studies have reported that bilateral THA has complication rates similar to those of unilateral THA [8, 11]. In Finland, bilateral THA made up approximately 1.4% of all THA performed between 2015 and 2020 [1].

Yoshii et al. [12] compared patient satisfaction, including pain, motion, and mental status, in bilateral THA and unilateral THA groups. They reported that patients who underwent bilateral THA showed greater improvements in motion and mental status compared to those with unilateral THA. This may be partly explained by the fact that in that study, patients who underwent bilateral THA had more severe symptoms preoperatively compared to those with unilateral THA. In a functional recovery study, no group differences between one-stage bilateral or unilateral THA in mobility, pain, and fear of movement were reported postoperatively after 3 and 7 days [13]. Berend et al. [14] did not find any differences between both patient groups in long-term patient survival, prosthetic survival, and functional outcomes. Moreover, no group differences in revision rates have been observed [8]. In recent study Turppo et al. [15] investigated self-reported physical capability and subjective well-being in long-term outcomes. They found that those outcomes were lower in women with arthroplasty compared controls without arthroplasty. Also, THA seemed to be more successful compared to TKA [15].

There have been studied complications [8, 11], short-term physical recovery after one week of operation [13], and patients´ satisfaction [12] between unilateral THA and simultaneous bilateral THA. Also, females self-reported physical capability with or without arthroplasties have been reported [15]. Many articles have compared to two-stage THA and simultaneous THA [9, 10]. However, there are a limited number of studies concerning differences in physical function between unilateral THA and simultaneous bilateral THA after long follow-up time. Therefore, it is important to study the long-term follow-up outcomes between these groups.

Our aim was to report patients’ pain and physical function after one- and five-years post-operatively among patients who underwent unilateral THA and those who underwent bilateral THA at the same time in one orthopaedic hospital in Finland.

## Methods

**Fig. 1:**
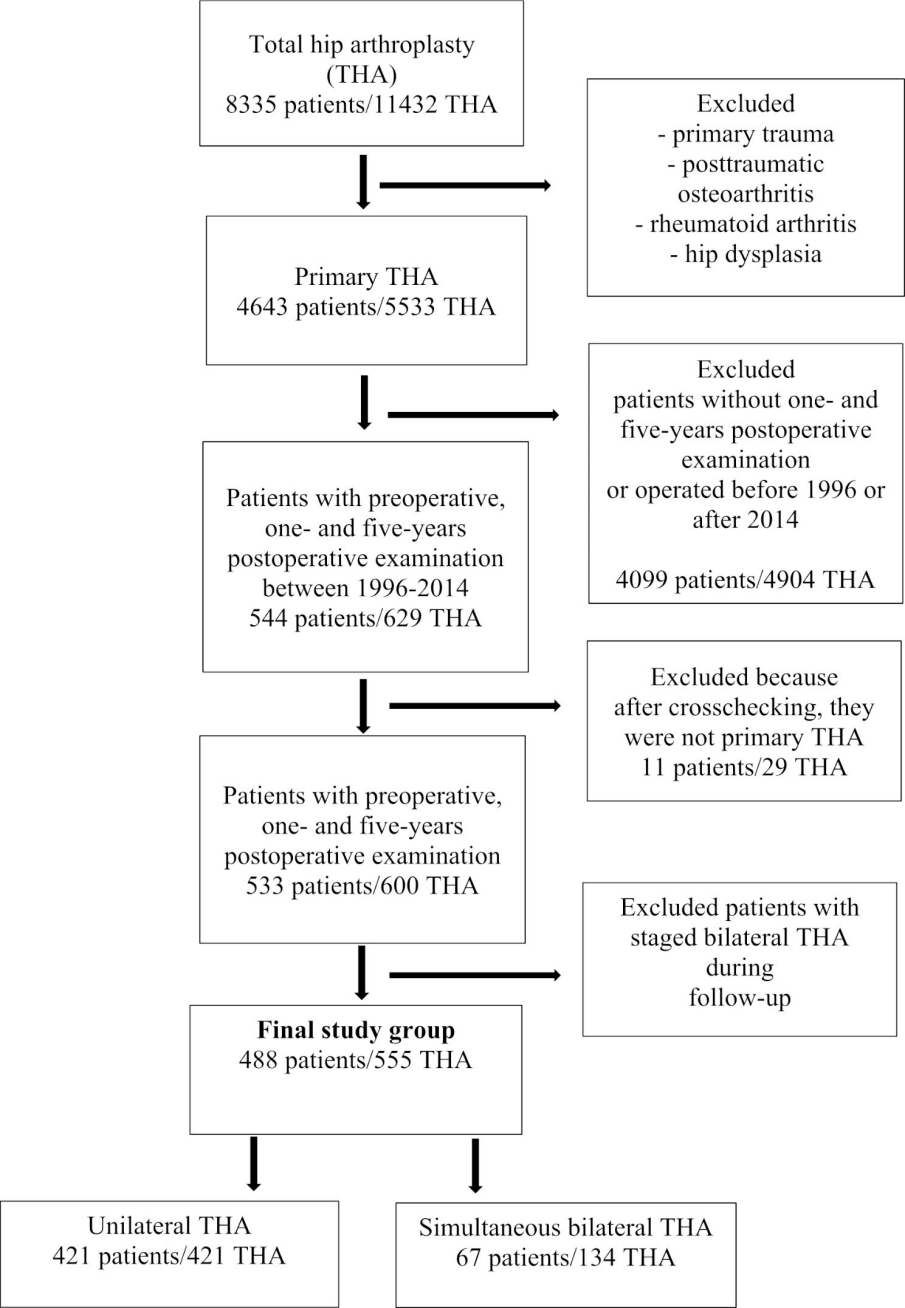
Flow chart of study group selection

Our study included patients who had THA between 1996 and 2014 at one orthopaedic hospital in Finland. We could have on average 5-years follow-up to the latest 2019. During these years, 7218 THAs were performed. Patients with primary hip osteoarthritis were included in this study (n = 3670/7218, 51%). Patients who were operated on due to primary trauma, posttraumatic OA, rheumatoid arthritis, or hip dysplasia were excluded. We also excluded patients who had another hip surgery during this follow-up period (two-staged THA, n = 45). Therefore, our sample included patients who underwent THA only in one hip or in both hips at once. Patients also had to have at least two orthopaedic clinical follow-up examinations after THA. The follow-up times were on average one year and on average 5 years after THA. The final study group consisted of 488 patients, and the total number of THA was 555. Unilateral THA was performed on 421 patients and simultaneous bilateral THA (later bilateral THA) was performed on 67 patients (Fig. 1). This is a retrospectively selected cohort study from one orthopaedic hospital and data was collected from patient records. Before 2000, bilateral THA was performed in 20 patients, and since then, it has been performed in 47 patients.

An orthopaedic surgeon performed the clinical examinations before the THA operation and after one and five years of operation and filled out the same structured form each time. The Charnley classification (one affected hip, two affected hips, others) was fulfilled, and leg-length inequality (LLI) (≥ 1 cm) was measured manually using the block method [16]. The patients rated their hip pain and physical function, such as walking distance, limping, use of walking aids (support), walking stairs, and ability to enter public transportation (Table S1). Pain was classified as four subsections such as no pain at all (40 points), mild pain after exertion (35 points), moderate pain (often painkillers) (20 points), severe pain (rest pain/severe motion pain) (0 points). After scaling the points were between 50 (no pain at all) and 0 (severe pain). Data were collected systematically using the “original Orton Hip Score” form to measure self-reported pain and physical function (Supplementary materials, Table S1 shows details of the collected data). We scaled our “original Orton Hip Score” (max 80 points) 0–100 points by multiplying the original Orton Hip Score points by 1.25 (100/80). This transformation is called the scaled Orton Hip Score (sOHS) in this study. In the additional table S1 can be seen the details of the scores, sOHS and Harris Hip Score (HHS), which has usually been used in THA [17] (Table S1).

Apart from our own hospital registry data of patients who underwent THA, we also received the register data of revision(s) and mortality events from the Finnish Institute of Health and Welfare.

### Statistical analyses

Summary statistics were described using mean and standard deviation (SD), median and interquartile range (IQR), or numbers as percentages. Statistical evaluation between THA (bilateral and unilateral) groups were analysed by using Student’s t-test, Mann–Whitney U test, Pearson’s chi-squared test, and Fisher’s exact test. Longitudinal data of the changes in mHHS were analyzed between the bilateral THA and unilateral THA groups with multilevel mixed-effects linear regression models using unstructured covariance structure. Mixed-effects models included main effects (group and time) and their interaction [18]. Time-to-event evaluation was based on the product limit estimate (Kaplan-Meier) of the cumulative function of the revision rate. Patients were followed until the end of the follow-up period, revision, or death. The revision rate functions between the two THA groups were compared by using the log-rank test of equality. Revision rates were presented as percentages and their 95 per cent confidence intervals. The Cohen’s effect-size statistics (*d*) calculated at five years after THA operation among the bilateral THA and unilateral THA groups. Effect size values of 0.20, 0.50, and 0.80 indicate small, moderate, and large, respectively [19]. 95 per cent confidence intervals (CIs) for the effect sizes were estimated by using bootstrapping method (10 000 replications) [20]. Hommel’s multiple comparison adjustment procedure (α level 0.05) was applied to correct the levels of significance [21]. The analyses were adjusted for age and gender, if necessary. In addition, 95% confidence intervals were presented for the most important results. Statistical analyses were performed using STATA software, version 17.0 (StataCorp LP; College Station, Texas, USA).

## Results

In the bilateral and unilateral THA groups, 54% and 58%, respectively, were women (Table 1). There were no differences between gender or age in these THA groups. Slightly over half of the operations (54%) were uncemented in the bilateral THA. The proportion of cemented, uncemented, or hybrid prothesis was different between the bilateral THA and unilateral THA groups (p = 0.002) (Table 1).

**Table 1:** Characteristics of patients in the simultaneous bilateral THA and unilateral THA groups

	Unilateral THA N = 421	Bilateral THA N = 67	P-value
Women/men, n (%)	244 (58)/177(42)	36 (54)/30 (46)	0.52
Age, mean (SD)	64 (11)	62 (9)	0.11
Charnley classification*, n (%)			.
One affected hip	295 (70)	0 (0)	
Bilateral hip affected	114 (27)	67 (100)	
Others	12 (3)	0 (0)	
Operation			0.002
Uncemented (NFB30)	125 (30)	36 (54)	
Hybrid (NFB40)	126 (30)	16 (24)	
Cemented (NFB50)	163 (39)	15 (22)	
Difficult primary THA (NFB60)	6 (1)	0 (0)	
scaled Orton Hip Socre (sOHS)**, mean (SD)			
Before operation	53 (20)	53 (19)	0.92
One year after operation	95 (9)	98 (4)	< 0.001
Five years after operation	95 (10)	97 (7)	0.003
Hospital visits, median (IQR)***	0 (0.2)	0 (0.3)	0.69

* Charnley classification

**scaled Orton Hip Score, the worst pain and physical function (0 points) and no pain and the best physical function (100 points); age and gender adjusted

*** Hospital visits, median and IQR = interquartile range

**Fig. 2:**
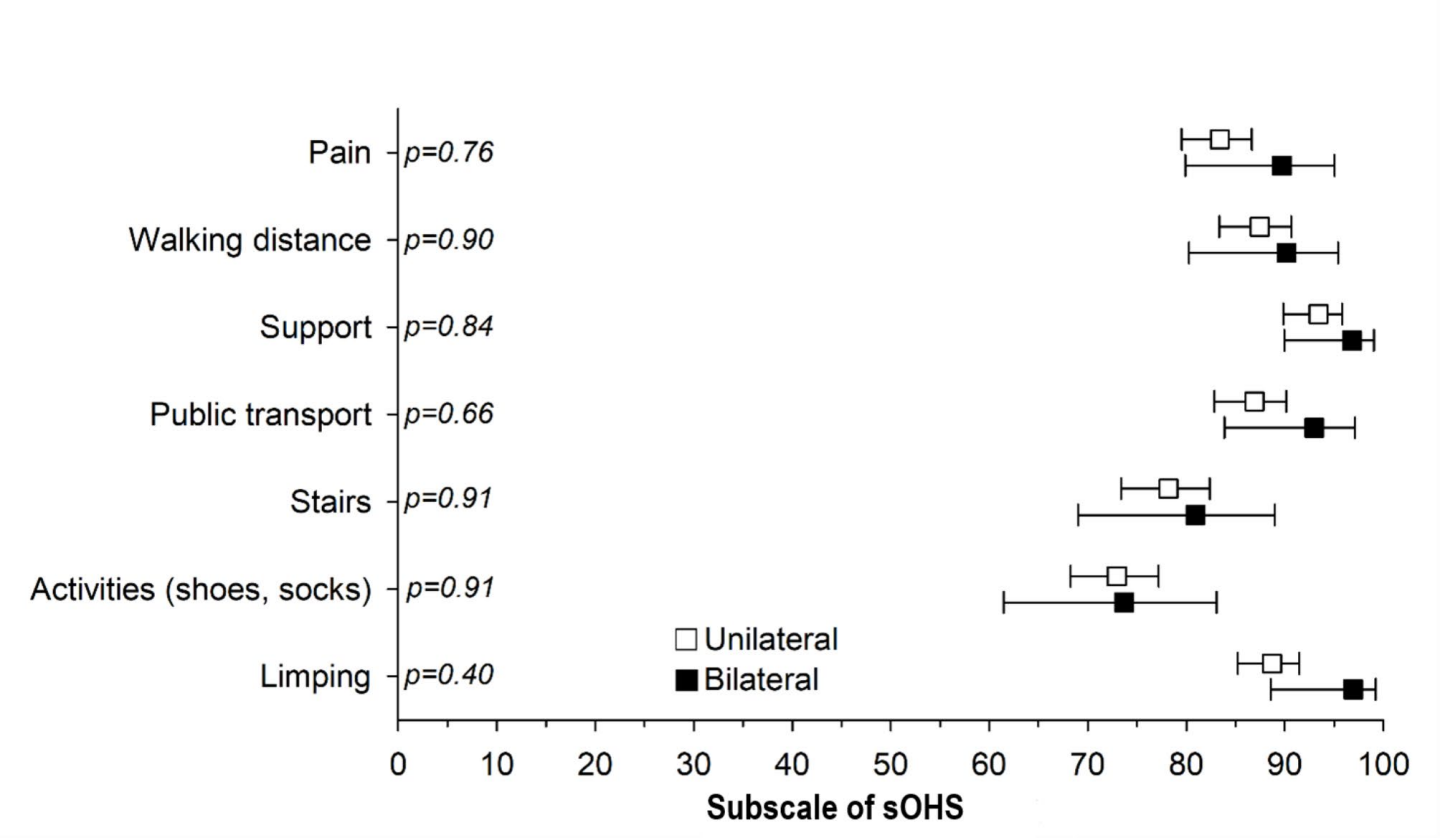
Age- and gender-adjusted subscales of the scaled Orton Hip Score (sOHS) in unilateral THA and bilateral THA after five years of total hip arthroplasty. Values with 95% confidence intervals (95% CI) were adjusted for age and sex. P-values were corrected using Hommel’s multiple comparison procedure

The mean sOHS before operation was 53 points out of 100 in both the unilateral and bilateral THA groups, no significant differences between the groups were found (Table 1). The preoperative sOHS was lower among women both in the unilateral and in the bilateral THA group than in men (mean 50 [SD 20] vs. 57 [SD 20], p = 0.001; 48 (SD 22) vs. 58 (SD 15), p = 0.004, respectively). At the one-year follow-up, sOHS improved remarkably from the preoperative situation both in the unilateral THA (age and gender adjusted mean improvement 42 points (95% confidence interval [95% CI]: 40 to 44) and in the bilateral THA groups (age and gender adjusted 45 (95% CI: 41 to 49), p = 0.30. At the five-year follow-up in both groups, sOHS remained, on average, almost the same level as sOHS at the one-year follow-up (Table 1). Patients who underwent bilateral THA had slightly higher total sOHS both after one- (98) and five-year’s (97) follow-ups compared to those who underwent unilateral THA (95, p < 0.001; 95, p = 0.003, respectively) (Table 1). However, at the five-year follow-up, no clinically significant differences were found between the patients who underwent unilateral THA and those who underwent bilateral THA (Fig. 2).

Based on the Charnley classification (one hip or two hips involved), 27% (N = 114) of the patients in the unilateral THA group were classified as having had both hips involved at baseline examination. This allowed us to compare the possible role of an involved, unoperated contralateral hip in recovery among unilateral THA patients. At baseline, the patients in the unilateral THA group with both hips involved reported more functional limitations than those with only one hip involved (age- and gender-adjusted mean total sOHS, 55 [SD 20] vs. 48 [SD 21], p = 0.006). One year after the operation in the unilateral THA subgroups, patients with one hip involved had higher total sOHS (mean 96 [SD 8]) than patients with both hips involved (mean 94 [SD 10], p = 0.027). The same trend was observed after five years of operation (mean 95 [SD 10] vs. 93 [SD 10], p = 0.016).

**Table 2:** Age- and gender-adjusted physical functions subscales at baseline, and five-year follow-up changes in the scaled Orton Hip Score (sOHS) among THA patients

		Baseline information		Change from baseline to five years after		
		(before the THA operation)		THA operation		
	Points	Unilateral THA	Bilateral THA	Unilateral THA	Bilateral THA	p-value*
	Range	Mean (SD)	Mean (SD)	Mean (95% CI)	Mean (95% CI)	
Pain	0–40	16 (11)	17 (10)	23 (22 to 24)	23 (20 to 26)	0.86
Walking distance	0–15	9 (4)	9 (4)	5 (4 to 5)	6 (5 to 7)	0.16
Limping	0–5	4 (1)	4 (1)	1 (0.9 to 1.1)	1 (1.0 to 1.5)	0.41
Support	0–5	4 (1)	4 (1)	1 (0.5 to 0.7)	0.6 (0.3 to 0.9)	0.86
Public Transport	0–5	3 (2)	3 (2)	1 (1.1 to 1.4)	2 (1 to 2)	0.28
Stairs	0–5	3 (1)	3 (1)	1 (1.1 to1.4)	1 (1 to 2)	0.85
Activities (shoes, socks)	0–5	2 (2)	2 (2)	2 (1.6 to 2.0)	2 (2 to 3)	0.66
Total sOHS (0-100)	0-100**	53 (20)	53 (19)	42 (40 to 44)	44 (40 to 49)	0.19

* p-values were adjusted for age and sex and corrected using Hommel’s multiple comparison procedure

** Total sOHS is scaled 0 to 100 (0 indicates the worst pain and physical function and 100 indicates no pain at all and the best physical function)

**Fig. 3:**
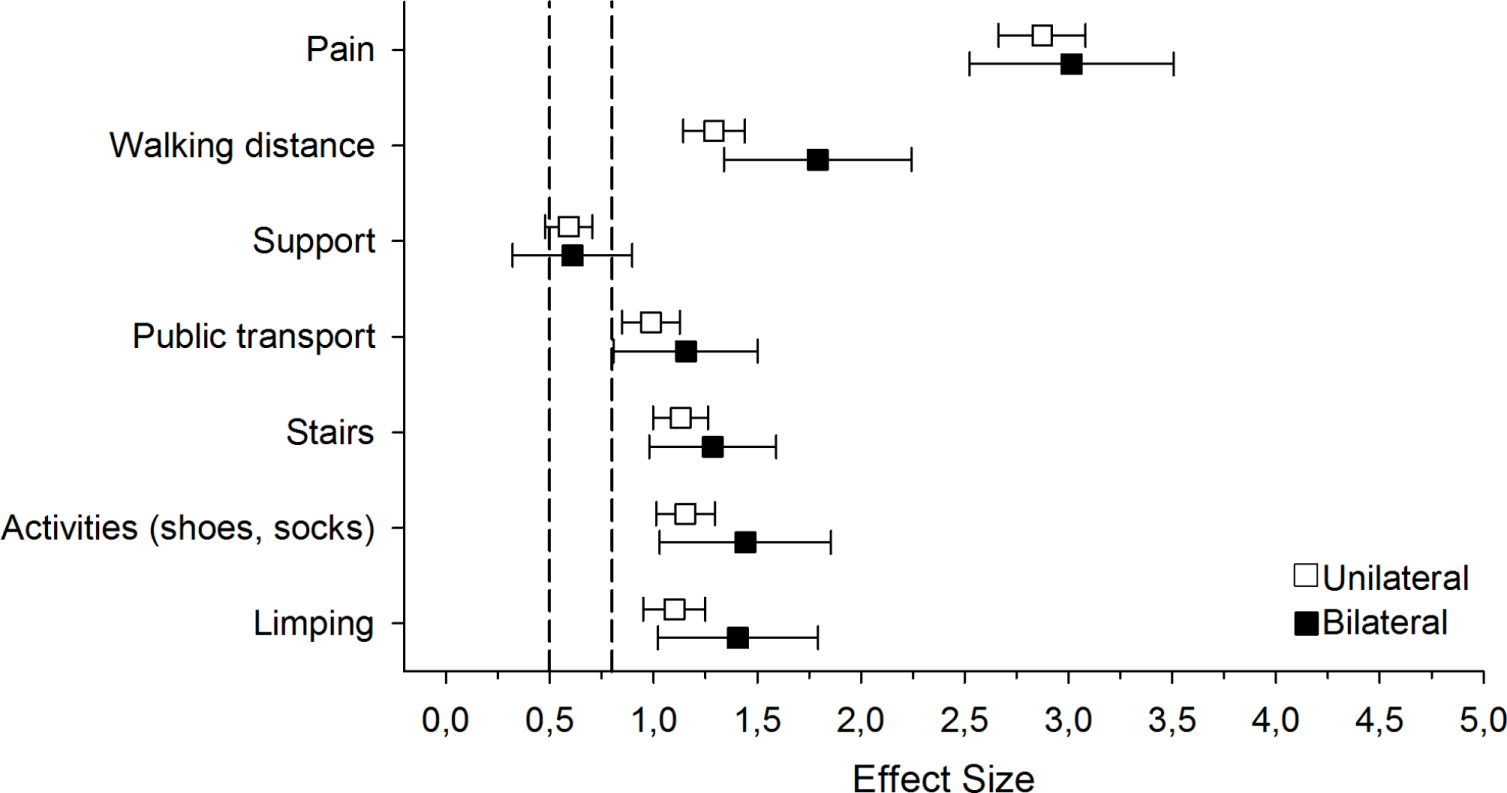
The effect sizes at five years after THA operation among the bilateral THA and unilateral THA groups in all pain and physical function subscales

Table 2 presents the sOHS at the baseline and changes during the five-year follow-up. Total baseline sOHS did not differ in the unilateral compared to the bilateral THA groups. After five years, the change in the total sOHS and its subscales was on the same level in both groups (Table 2). The effect sizes five years after the THA operation were large (over 0.8) among the bilateral THA and unilateral THA groups in all pain and physical function subscales, except for using walking aids, whose effect size was moderate (between 0.5 and 0.8) (Fig. 3). No differences were observed between the groups regarding the effect sizes.

LLI (≥ 1 cm) was more common at one-year follow-up among unilateral THA than among bilateral THA patients (14% vs. 4%, p = 0.001). Further, patients who underwent unilateral THA and had LLI reported more functional limitations than those who underwent unilateral THA without LLI after one year of operation (total sOHS mean 91 [SD 12] vs. 96 (SD 8), p < 0.001).

To investigate the revision rate, we were able to use a five-year follow-up period. During this follow-up period, seven patients died. The revision rate during the follow-up was 1.4% (95% CI: 0.6 to 3.2) among patients who underwent unilateral THA and 1.5% (95% CI: 0.2 to 10.1) among those who underwent bilateral THA (p = 0.67).

## Discussion

Our registry study showed that physical function improved remarkably one year after the operation, both in patients who underwent bilateral THA and those who underwent unilateral THA. This improved function and physical ability was maintained, on average, five years after the operation in both groups. Patients’ pain decreased and physical function improved, measured by sOHS, in both groups. Patients in the bilateral THA group had, on average, higher total sOHS values than those in the unilateral THA group during the one-year and five-year follow-ups. The clinical meaning of such a difference is small, and no group differences exist in the sOHS subscales of pain and physical function subscales.

Nowadays, THA represents a considerable part of the orthopaedic routine. In the literature, both bilateral THA and unilateral THA have similar complication risks [6, 22]. Short-term functional results were reported by Temporiti et al. [13]. Assessments were performed before surgery and three and seven days postoperatively in both groups. As expected, patients’ physical function was worse in both groups after seven days of operation compared to the situation before the operation. The researchers also found a tendency for slightly more pain among bilateral THA patients than among unilateral THA patients [13].

In Yoshii et al.’s [12] one-year follow-up study, motion, and mental health improvement rates were higher in the bilateral THA group than in the unilateral THA group. However, pain level reduction was similar in both groups [12]. Aghayev et al. [9] reported that walking distance after operation was better in patients who underwent bilateral THA than in those who underwent unilateral THA. However, their study compared simultaneous bilateral THA and two-stage THA procedures. In their study of only patients who underwent unilateral THA, Goeb et al. [23] found a significant correlation between increased weekly steps and improved scores (Hip Disability and Osteoarthritis Outcome Score-Junior) after six weeks of THA. In the current study, walking distance was divided into five categories (not able to walk at all – walking distance more than 1.5 km). The patients’ walking distance was improved to being able to walk over 1.5 km after one year of THA in both groups. Patients who underwent bilateral THA could walk long distances more often. By contrast, in their long-term (27-year) follow-up study, Berend et al. [14] found no differences between bilateral THA and unilateral THA groups and HHS. A register-based study [9] reported that patients with bilateral THA were, on average, six years younger than unilateral THA patients. In both patients who underwent bilateral or unilateral THA, functional outcome was shown to also deteriorate after surgery in young patients [13]. In our study, one year after bilateral THA, physical function improved in patients, even more than in patients who underwent unilateral THA. There were no differences between the age, gender, and THA groups.

We specifically examined the pain and physical functions subscale (sOHS) in patients before the operation. Our results are in line with the earlier view that women have, on average, significantly lower HHS than men preoperatively [24]. However, after THA, no differences between genders were seen in sOHS, similar to Aghayev et al.’s [9] report. Kostamo et al. [24] found a lower HHS in women than in men after THA. However, the change between preoperative HHS and postoperative HHS was not different between the genders in their study.

Among patients who underwent unilateral THA, we observed that patients with only one hip involved before operation had better total sOHS after one- and five-years of operation compared to those both hips involved. When both hips are involved, there can be more pain in the hips, and physical function, such as walking and daily activities, can be worse compared to patients with only one hip involved. Röder et al. [25] included patients who underwent unilateral THA and an untreated but affected contralateral hip. They concluded that patients with bilateral THA had significantly better walking capacities than patients with unilateral THA and an untreated contralaterally diseased hip. Achieving better physical function when both hips involved may be one factor that explains why both hips are treated operatively at the same time.

The effect size of change was large among both groups in almost every pain or physical function subscale. Although we calculated the change in using walking aids from pre-operation to long-term follow-up, the moderate effect size may be partly explained by the fact that about 60% of the patients did not use any walking aids before the operation. In Ayekoloye et al.’s [26] studied long-term survival following conversion of hip fusion to THA. 31% of patients required the use of walking aids preoperatively and after on average 12 years follow-up, 68% of patients used the walking aids, especially walking long distances. Same trend was seen in Nilsdotter and Isaksson [27] study, where seven years after THA 46% of patients need of walking aids. In our study, the use of walking aids in all patients was 40% preoperatively and 9% after one year and 12% after five years of operation. Patients who had one hip operated had more common need of walking aids preoperatively than patients who had both hips operated. During the follow-ups no differences between requiring walking aids and these groups were found.

In the present study, the revision rate and mortality rates were small in both groups. No differences were found between the rates and groups. In the Finnish registry data 2020, the revision rate was approximately 4% after five years of primary THA [1]. In a recent study by Loppini et al. [28], the reoperation rates were 0.7% in unilateral THA and 0.3% in bilateral THA at 30 days, and 0.9% and 1.8% at 1 year, respectively, percentages that are nearly similar to those in our study after 5 years of follow-up. Stavrakis et al. [8] reported a minor number of revisions both in patients who underwent bilateral and those who had unilateral THA.

Based on a retrospective study by Micicoi et al. [11], patients who underwent bilateral THA had LLI less often than those who underwent unilateral THA. LLI of ≥ 1 cm was also more common in the unilateral THA group than in the bilateral THA group in our study. White and Dougal [29] did not find a statistical association between LLI after six months of THA operation and functional outcome or patient satisfaction. In our study, unilateral THA patients with LLI over one centimetre had lower total sOHS in the one-year follow-up than unilateral THA patients without LLI. In line with our study, Zhang et al. [30] reported that HHS was higher in patients without LLI after one year of follow-up.

Patients who are selected for bilateral THA operations are often relatively young and do not have comorbidity with severe symptoms [31]. This may partly explain the good recovery and physical function after THA. However, patients with unilateral THA may also have pain, restricted range of motion, and other symptoms in their non-operated hip, restricting activities of daily living. We more carefully examined our unilateral THA group and compared its subgroups: one or two hips involved. We found that patients who underwent unilateral THA preoperatively with only one hip involved had better physical function (total sOHS score) before operation, which became better during follow-ups. It may be that the recovery would be worse if both hips were painful and restricted patients’ physical function before the operation. Careful patient selection can further minimise the risks of bilateral THA and increase the likelihood of consistently successful outcomes [32]. It has been stated that bilateral THA is related to high patient satisfaction and high safety [12].

There are some limitations to this study. The study group was taken from one orthopaedic hospital. The patients had typically participated in follow-up clinical examinations with commitment, which might have improved their participation rates. However, those patients who were satisfied with the outcome may have been lost to follow-up. Overall, the influence of participation bias is unknown. Further, our data were systematically gathered in the same way during 1996–2014. However, when the data collection began in 1996, some HHS questions were not included for unknown reasons. Therefore, we used a sOHS. On the one hand, this restricts the comparison of our results to other earlier studies. On the other hand, a structured, long-lasting follow-up system ensured the comparability of the bilateral and unilateral THA groups. In their study of modified HHS, Vishwanathan et al. [33] concluded that it has adequate construct validity and responsiveness to evaluate the functional outcome of fixation in pertrochanteric hip fractures. Lastly, the data used in the present study have been systematically collected since 1987. However, data for this follow-up study of THA operations was obtained from 1996, which allowed the World Health Organization’s ICD-10 disease classification, our data, and the registry data from the National Institute of Health and Welfare to be comparable.

## Conclusions

Our findings showed that both unilateral THA and bilateral THA groups had increased physical function, and decreased pain after one year follow-up of the primary THA operation. The outcomes were almost the same after five years of follow-up. Patients who underwent bilateral THA had slightly better physical function than patients with unilateral THA at follow-up. However, this outcome was not associated with any clinical relevance.

### Electronic supplementary material

Below is the link to the electronic supplementary material.

Supplementary Material 1

## Data Availability

The datasets generated and analysed during the current study are not publicly available because they were retrospectively obtained from institutional registries and records that are not available for an open access repository. The data are available from the corresponding author upon reasonable request.

## Abbreviations

CI: confidence interval
IQR: interquartile range
HHS: Harris Hip Score
LLI: leg-length inequality
sOHS: scaled Orton Hip Score
SD: standard deviation
THA: total hip arthroplasty
