# Correction: Simultaneous bilateral versus unilateral total hip arthroplasty: pain and physical function in a one- and five-year follow-up - retrospective patients record study

**DOI:** 10.1186/s12891-023-06809-9

**Published:** 2023-09-05

**Authors:** Leena Ristolainen, Jyrki Kettunen, Jouni Lohikoski, Hannu Kautiainen, Mikko Manninen

**Affiliations:** 1 Orton Orthopaedic Hospital, Tenholantie 10, Helsinki, 00280 Finland; 2 Arcada University of Applied Sciences, Jan-Magnus Janssonin aukio 1, Helsinki, 00550 Finland; 3 Primary Health Care Unit, Kuopio University Hospital, P.O. Box 100, Kuopio, FI 70029 KYS Finland

**Correction:** ***BMC Musculoskelet Disord*** **24, 608 (2023)**

10.1186/s12891-023-06743-w

Following publication of the original article [1], the authors would like to correct the names of all authors. The given name and family name were reversed.

**The correct author names are (first name, last name)**:

Leena Ristolainen

Jyrki Kettunen

Jouni Lohikoski

Hannu Kautiainen

Mikko Manninen

The author group has been updated above and the original article [1] has been corrected.
