# Supplementary material for: Simultaneous bilateral versus unilateral total hip arthroplasty: Pain and physical function in a one- and five-year follow-up - retrospective patients record study
Source: BMC Musculoskelet Disord. 2023 Jul 25;24:608. doi: 10.1186/s12891-023-06743-w (PMC10367357; doi:10.1186/s12891-023-06743-w)
Supplement: Supplementary file 1 — Supplementary Material 1 [file 12891_2023_6743_MOESM1_ESM.docx]

Table S1. Original “Orton Hip Score”, scaled Orton Hip Score (sOHS) in our study scaled to the Harris Hip Score.

| **Original Orton Hip Score  (points)** | | **scaled Orton Hip Score (sOHS)**  **(points), (this study)** | | **Harris Hip Score (points)**  **(Harris, 1969) (17)** | |
| --- | --- | --- | --- | --- | --- |
| Pain (40/35/20/0) | | Pain (50/43.75/25/0) | | Pain (44/40/30/20/10/0) | |
| Function: Gait | | Function: Gait | | Function: Gait | |
|  | Support (5/4/3/2/1/0) |  | Support (6.25/5/3.75/2.5/1.25/0) |  | Support (11/7/5/3/2/0) |
|  | Walking distance (15/12/7/2/0) |  | Walking distance (18,75/15/8,75/2,5/0) |  | Walking distance (11/8/5/2/0) |
|  | Limp (5/4/2/0) |  | Limp (6.25/5/2.5/0) |  | Limp (11/8/5/0) |
| Function: Activities | | Function: Activities | | Function: Activities | |
|  | Stairs (5/4/2/0) |  | Stairs (6.25/5/2.5/0) |  | Stairs (4/2/1/0) |
|  | Activities – shoes/socks (5/3/0) |  | Activities – shoes, socks (6.25/3.75/0) |  | Activities – shoes/socks (4/2/0) |
|  | Public transportation (5/3/0) |  | Public transportation (6.25/3.75/0) |  | Public transportation (1/0) |
|  |  |  |  |  | Sitting (5/3/0) |
|  |  |  |  | Absence of deformity (4/0) | |
|  |  |  |  | Range of motion (5) | |
|  |  |  |  |  | Flexion (3.9–0) |
|  |  |  |  |  | Abduction (0.65–0) |
|  |  |  |  |  | External rotation (0.3–0) |
|  |  |  |  |  | Adduction (0.15–0) |
| **Total 80** | |  | **100** |  | **100** |

Orton Hip Score: 0 points = worst pain and disability to 80 points = no pain and no disability.

scaled Orton Hip Score and Harris Hip Score: 0 points = worst pain and disability to 100 points = no pain and no disability.
